# Prophylactic HIPEC in pT4 Colon Tumors: Proactive Approach or Overtreatment?

**DOI:** 10.1245/s10434-019-07970-z

**Published:** 2019-10-29

**Authors:** Nerea Borda Arrizabalaga, José María Enriquez Navascués, Garazi Elorza Echaniz, Yolanda Saralegui Ansorena, Carlos Placer Galán, Xabier Arteaga Martín, Leyre Velaz Pardo

**Affiliations:** Donostia University Hospital, Donostia-San Sebastian, Spain

## Abstract

**Background:**

The peritoneum is the second most common site for metastasis in patients with colorectal cancer. Various factors have been studied to identify patients at risk of developing peritoneal carcinomatosis (PC), including T4 tumors. The objectives were to assess the incidence of synchronous and metachronous PC, explore potential risk factors for developing PC as the only site of metastasis, and identify which patients might be candidates for prophylactic hyperthermic intraperitoneal chemotherapy (HIPEC).

**Methods:**

We conducted a retrospective analysis of 125 patients with pT4 colon cancer who underwent surgery in a single center between January 2010 and December 2014.

**Results:**

Of the 947 colon cancer patients who underwent surgery, 125 (13.2%) were diagnosed with pT4a or b colon carcinoma. The median follow-up was 3.7 years. The overall rate of PC was 34.3%, being synchronous in 12% and metachronous in 22.3% of cases. The 8% and 6% of synchronous and metachronous cases of PC respectively were isolated (single site) metastasis. The incidence of PC was 6.1% at 1 year and 14.5% at 3 years after surgery. pT4 was not found to be an independent risk factor for the development of PC (*p* = 0.231). Nonetheless, the rate of metachronous PC as a single site of metastasis was higher in patients with pT4 tumors and peritoneal nodules around the primary tumor and/or tumor perforation (*p* = 0.027) and/or who underwent emergency surgery (*p* = 0.043) than other patients.

**Conclusions:**

Considering pT4 tumor stage as the only risk factor for the development of PC in deciding whether to administer prophylactic HIPEC would lead to unjustified overtreatment.

Worldwide, colorectal cancer is the third most common cause of cancer in men and the second in women. It is expected to affect some 2.4 million individuals by 2034.^1^ The peritoneum is the second most common site of metastasis in patients with colorectal cancer, accounting for 25–35% of all cases of recurrence.^2^^,^^3^ Among patients with recurrent disease, 5–10% have synchronous and 20–50% develop metachronous peritoneal carcinomatosis (PC).^4^–^7^

The treatment for PC has evolved greatly over the past 15 years. The goal of the treatment has changed from being purely palliative or supportive to being considered curative in selected patients. The combination of cytoreductive surgery (CRS) and hyperthermic intraperitoneal chemotherapy (HIPEC) has achieved median survival rates of up to 64 months; peritoneal cancer index (PCI) and complete surgical cytoreduction (CRS-0) are the two main predictors of prognosis with this strategy.^8^–^15^ Nonetheless, CRS and HIPEC are not risk-free, with nonnegligible associated morbidity and mortality.^16^,^17^

Numerous clinical, pathological, and biological factors have been studied to identify patients with the highest risk of having synchronous or developing metachronous PC and to be able to diagnose it early, while the PCI is still low, and have an impact on outcomes in this population.^18^–^20^ With the same goal of altering the natural history of PC, two different strategies have been proposed: one consisting of administering prophylactic HIPEC at the time of primary tumor surgery to prevent peritoneal recurrence, and the other performing systematic second-look surgery with HIPEC, preempting peritoneal recurrence.^18^

In addition to spontaneous or iatrogenic tumor perforation, peritoneal tumors around the primary tumor and ovarian metastases, the presence of tumors at stage T4 is a potential risk factor for the development of PC.^18^ It has been estimated that up to 26% of patients with T4 tumors would benefit from either of the aforementioned strategies.^21^ Nonetheless, the real incidence of PC as an isolated finding in stage T4 disease is debated and we may be overestimating the problem. On the one hand, preoperatively, based on imaging alone, T4 tumors may be overdiagnosed, and on the other, if the incidence of metachromous PC were lower than expected use of the prophylactic strategies would result in overtreatment, with the associated morbidity.

The primary objective of this study was to assess the incidence of synchronous and metachronous PC in pT4 colon cancer patients who underwent surgery and were followed-up in an integrated healthcare organization, with medical records and responsibility for patient monitoring shared between hospital and primary care providers. The secondary objectives were to explore potential risk factors for the development of PC as the only site of metastasis and identify which patients might be candidates for prophylactic HIPEC.

## Methods

We performed a retrospective analysis of pT4 colon cancer patients, regardless of whether they had lymph node involvement and/or distant metastasis, who underwent surgery between January 2010 and December 2014 in a healthcare region with integrated social and primary and specialist health care, with a shared follow-up protocol, and with no missing data. The study protocol was approved by the scientific research ethics committee of the institution.

The TNM cancer staging was based on the Seventh Edition of the American Joint Committee on Cancer Colon and Rectum Cancer Staging Manual. Accordingly, T4a tumors were defined as those with serosal involvement and T4b tumors as those in which the tumor has spread to neighboring organs.

We recorded data on epidemiological, clinical, surgical, and histopathological characteristics, the initial staging and findings during follow-up. The metachronous PC was diagnosed by imaging tests: abdominal ultrasound and CT scans. These tests were performed every 6 months (at least one CT scan per year) for the first 3 years and annually until the 5-year mark. Criteria for establishing metachronous PC included: histologic or cytologic confirmation, palpable disease, disease evident on radiographic studies with subsequent image or clinical progression, and supportive biochemical data, i.e., rising level of carcinoembryonic antigen (CEA). No patient was accepted as having PC by virtue of one interval CT, alone. A colonoscopy was performed at 3 years after the surgical intervention (or at 1 year if no previous colonoscopy had been complete).

### Statistical Analysis

Qualitative data were expressed as percentages and absolute values and compared with the *χ*^2^ test. In the case of quantitative variables, first, the Kolmogorov–Smirnov test was used to characterize the distribution, and then data were expressed as means and standard deviations and compared using Student’s *t* test if normally distributed, and otherwise, they were expressed as medians and interquartile ranges and compared using the Mann–Whitney *U* test.

Data on overall survival, disease-free survival and time to progression were analyzed using Kaplan–Meier curves, and factors were compared using log-rank tests. The variables with a significance level ≤ 0.2 were included in the binary logistic regression to explore which were associated with prognosis including COX-2.

Survival was measured from the date of the surgical intervention for the primary tumor until the date of the last follow-up or until the patient died. *p* values < 0.05 were considered statistically significant.

## Results

A total of 947 patients underwent colon cancer surgery at our institution between January 2010 and December 2014. Of these, 125 patients (13.2%) were diagnosed with pT4a or b colon carcinoma. Data were collected until 31 December 2017, with a median follow-up of 3.7 (range 1–7.7) years.

The main clinical characteristics of the patients and histopathological characteristics of the tumors are described in Table 1. As can be observed, a quarter (25.6%) of patients had PC, distant metastasis, or both at the time of diagnosis. Overall, 12% had synchronous PC, 8% corresponding to patients with isolated dissemination, and 4% to patients who also had distant metastasis. Having a primary tumor that has spread to neighboring organs (pT4b) and lymph node involvement were identified as risk factors for synchronous PC (Table 2).

**Table 1:** Clinical characteristics of patients and histopathological characteristics of the tumors

Mean age	67 years old (32–91)
Sex	
Male	71 (56.8)
Female	54 (43.2)
Site of primary tumor^a^	
Left colon	62 (49.6)
Transverse colon	10 (8.0)
Right colon	53 (42.4)
Type of surgery	
Scheduled	96 (76.8)
Emergency	29 (23.2)
Spontaneous/Iatrogenic perforation of the tumor	
No	88 (70.4)
Yes	37 (29.6)
Obstruction of the primary tumor	
No	98 (78.4)
Yes	27 (21.6)
Type of resection	
R0	110 (88)
R1	4 (3.2)
R2	11 (8.8)
Degree of tumor differentiation	
G1	36 (28.8)
G2	70 (56.0)
G3	19 (15.2)
Histological findings	
Adenocarcinoma	110 (88.0)
Mucinous adenocarcinoma	10 (8.0)
Signet ring cell carcinoma	5 (4.0)
pT4	
pT4a	81 (64.8)
pT4b	44 (35.2)
pN	
pN0	52 (41.6)
pN1	44 (35.2)
pN1a	19 (15.2)
pN1b	24 (19.2)
pN1c	1 (0.8)
pN2	29 (23.2)
pN2a	17 (13.6)
pN2b	12 (9.6)
M	
M0	93 (74.4)
M1	32 (25.6)
Tumor stage	
IIa	28 (22.4)
IIb	19 (15.2)
IIIb	24 (19.2)
IIIc	23 (18.4)
IVa	23 (18.4)
IVb	8 (6.4)
Anastomotic leakage	
No	113 (90.4)
Yes	12 (9.6)
Site of metastasis at primary tumor diagnosis	32 (25.6)
Visceral metastases	17 (13.6)
Peritoneal carcinomatosis	10 (8.0)
Visceral metastases + peritoneal carcinomatosis	5 (4.0)

^a^Left colon: sigmoid colon, descending colon, splenic flexure; right colon: caecum, ascending colon, hepatic flexure

**Table 2:** Risk factors for synchronous peritoneal carcinomatosis (univariate analysis)

	Peritoneal carcinomatosis (%)	*p*
Site of the primary tumor^a^		0.482
Left colon	6/62 (9.7)	
Transverse colon	0/9 (0.0)	
Right colon	7/54 (12.3)	
Degree of tumor differentiation		0.255
G1	3/36 (8.3)	
G2	6/70 (8.7)	
G3	4/19 (21.1)	
Transmural involvement (pT)		**0.028**
Serosal involvement	12/81(14.8)	
Spread to neighboring organs	1/44(2.3)	
pN		**0.026**
pN0	2/52 (3.8)	
pN1	3/19 (15.8)	
pN1a	3/24 (12.5)	
pN1b	1/1 (100)	
pN1c	2/17 (11.8)	
pN2	1/12 (8.3)	
pN2a		
pN2b		
Histological findings		0.473
Adenocarcinoma	12/116 (10.3)	
Mucinous adenocarcinoma	0/5 (0.0)	
Signet ring cell carcinoma	1/4 (25.0)	

^a^Left colon: sigmoid colon, descending colon, splenic flexure; right colon: caecum, ascending colon, hepatic flexure

Bold values are statistically significant (*p* < 0.05)

Table 3 summarizes the course of patients during the follow-up period. A total of 21 patients (22.3%) were found to have PC during follow-up, but only 6 (6.12%) had isolated PC, as a single site of recurrence, being the rates of PC at 1 and 3 years after surgery 6.1% (95% confidence interval [CI] 87.9–97.6%) and 14.5% (95% CI 76.6–91.2%), respectively. During the follow-up, tumor markers increased in 12 of 21 patients (57.2%), and in patients with metachronous PC as the single site of metastasis, increased CEA was observed in 4 of 6 (67%). Most of the patients were asymptomatic. Only three patients presented colic abdominal pain or lumbar pain. The PC was confirmed by surgery or biopsy in only four patients (19.1%).

**Table 3:** Follow-up

	N (%)
Development of distant metastases (no PC)	
No	72 (73.4)
Yes	26 (27.7)
Site of metastases	
Liver	14 (53.8)
Lung	9 (34.6)
≥ 2 organs	3 (11.5)
PC	
No	77 (78.6)
Yes	21 (22.3)
PC as a single site of metastasis	6 (6.1)
Local recurrence	
No	77 (78.6)
Yes	21 (21.4)
Mortality	63 (64.3)
Causes of mortality	
Progression of disease	42 (66.7)
Post-surgical complications	5 (7.8)
Comorbidities	16 (25.4)

*PC* peritoneal carcinomatosis

The potential risk factors for PC are summarized in Table 4. Among the variables analyzed, emergency rather than scheduled surgery (*p* = 0.019), tumor perforation (*p* = 0.047), and distant metastasis at diagnosis of the primary tumor (*p* = 0.047) emerged as independent risk factors for the development of metachronous PC, but they did not remain significant in the multivariate analysis (Table 5).

**Table 4:** Risk factors for developing metachronous peritoneal carcinomatosis (univariate analysis)

	Peritoneal carcinomatosis (%)	*p*
Type of resection		0.713
R0	19/110 (17.3)	
R1	1/4 (25.0)	
R2	1/11 (9.1)	
Degree of tumor differentiation		0.697
G1	7/36 (19.4)	
G2	12/70 (17.1)	
G3	2/17 (11.8)	
Histological finding		0.756
Adenocarcinoma	18/110 (16.36)	
Mucinous adenocarcinoma	2/10 (20.0)	
Signet ring cell carcinoma	1/5 (20.0)	
Transmural involvement (pT)		0.231
pT4a	16/81 (19.7)	
pT4b	5/44 (11.4)	
Lymph node involvement (pN)		0.235
pN0	6/52 (11.5)	
pN1	4/19 (21.1)	
pN1a	4/24 (16.7)	
pN1b	1/1 (100)	
pN1c	3/17 (17.6)	
pN2	3/12(25)	
pN2a		
pN2b		
Metastasis (M)		**0.047**
M0	12/93 (12.9)	
M1	9/32 (28.1)	
Tumor stage		0.371
Iia	2/28 (7.1)	
Iib	2/19 (10.5)	
IIIb	2/24 (8.3)	
IIIc	3/23 (13)	
Iva	6/23 (26.1)	
Ivb	6/8 (75)	
Type of surgery		**0.019**
Scheduled	12/96 (12.5)	
Emergency	9/29 (31.0)	
Spontaneous/iatrogenic tumor perforation	10/37 (27.1)	**0.047**
Primary tumor obstruction	6/27 (22.2)	0.395
Anastomotic leakage	0/12 (9.4)	0.102
Site of primary tumor^a^		0.827
Left colon	11/62 (17.7)	
Transverse colon	2/10 (20)	
Right colon	8/53 (15.1)	
Peritoneal implants around the primary tumor	5/125 (4.0)	0.157

^a^Left colon: sigmoid colon, descending colon, splenic flexure; right colon: caecum, ascending colon, hepatic flexure

Bold values are statistically significant (*p* < 0.05)

**Table 5:** Multivariate analysis exploring potential risk factors for the development of metachronous peritoneal carcinomatosis (N = 125)

	*p*	95% CI
Emergency surgery	0.052	0.168–1.01
Affectation of neighboring organs	0.992	0.295–3.848
pN	0.948	0.198–11.001
pM	0.109	0.824–6.770
Site of the primary tumor	0.799	0.246–2.942
Primary tumor perforation	0.118	0.168–1.221
Primary tumor obstruction	0.908	0.164–5.001
Peritoneal implants around the primary tumor	0.186	0.007–2.637
Anastomotic leakage	0.999	ns

*ns* no significance

Nonetheless, in the subgroup analysis, patients with pT4 colon tumors with perforation who underwent emergency surgery were at greater risk of developing PC than other patients (*p* = 0.043), as were patients with pT4 tumors with perforation and peritoneal nodules around the primary tumor (*p* = 0.027).

The 3- and 5-year overall survival rates were 60% (95% CI 50.8–67.8%) and 50.3% (95% CI 40.9–59%), respectively, with a median survival of 5.4 years. The mortality rate was 14 deaths/100/year. Five patients (7.8%) died in the postoperative period (2–3 months) due to complications related to the surgical intervention, 42 (66.7%) died due to disease progression, and 16 (25.4%) died due to other causes, namely, patient-related comorbidities.

## Discussion

In our study, the overall rate of PC in patients with pT4 colon carcinoma was 34.3%: 12% corresponding to synchronous and 22.3% to metachronous dissemination, respectively. On the other hand, the dissemination was only isolated (a single site) in 8% and 6% of cases of synchronous and metachronous PC, respectively. The incidence rate of PC was 6.1% at 1 year and 14.5% 3 years after surgery.

The overall incidence of PC in our series was higher than that in other studies, which have found rates of PC of 12–18% in patients with pT4 tumors^4^,^22^; however, despite complete clinical follow-up, the incidence of PC as a single site of metachronous metastasis was lower (6.1%) than in other research, which has indicated isolated PC in up to a third of patients, with rates of local and peritoneal recurrence of 15.6% and 36.7% at 1 and 3 years after surgery for the primary tumor, respectively.^21^ Notably, the overall survival in our series is similar to that found previously in consecutive nonselected patients with pT4 colon cancer.^21^ This leads us to believe that the series analyzed is representative of the tumor stage.

Procedures for diagnosing PC in the initial stages have considerable limitations. This shortage of effective tools for the early diagnosis of PC may result in an under-diagnosis of PC in cases in which systematic laparotomy/laparoscopy is not performed.

In our study, involvement beyond the serosa and involvement of lymph nodes at the time of primary tumor diagnosis have been identified as potential risk factors for synchronous but not metachronous PC. Regarding the development of metachronous PC, our univariate analysis suggests that emergency surgery and primary tumor perforation may be risk factors, although these findings were not confirmed in the multivariate analysis, likely due to the small sample size. Other possible risk factors directly related to the development of PC after curative colorectal surgery include peritoneal implants around the primary tumor, ovarian metastases, the primary tumor being in the right colon, and pT4 and pN2 stage disease.^23^,^24^

Although the prophylactic (surgery for the primary tumor + HIPEC) and preemptive (systematic second-look surgery + HIPEC) strategies are different, they have the common objective of improving oncological outcomes in patients at high risk of developing PC. Prophylactic HIPEC has the great appeal of not requiring an additional intervention, with potential further peritoneal and visceral resections,^17^ but it does have some downsides. On the one hand, it is not available at all times or in all centers offering surgery for colon cancer, and there is a risk of overtreatment, because it is not possible to identify the involvement of structures or differentiate between cT3 and cT4 intraoperatively. On the other hand, it has some ethical limitations, related to the lack of information and prior patient consent (oophorectomy, complications associated with an experimental treatment that is not free of complications, etc.). These logistic and ethical limitations have led to a clinical trial of the prophylactic strategy being withdrawn before completion of recruitment.^25^

Second-look surgery with HIPEC seems to be more accessible and appropriate for daily clinical practice. There are at least two currently ongoing clinical trials of preemptive strategies that differ not only in the selection of cases in relation to risk factors for the development of PC but also the optimal timing of the intervention. In the French trial (PROPHYLOCHIP), second-look surgery is performed 6–12 months after surgery for the primary tumor.^26^ In contrast, in the Dutch trial (COLOPEC), the second-look procedure is performed immediately after surgery for the primary tumor (10 days) or after an interval of 5–8 weeks.^20^ In both studies, there is consensus on performing this type of intervention in patients with isolated PC and various different risk factors for the peritoneal development, such as those mentioned above, but what about pT4 tumors?

Unexpectedly, both studies confirm that criteria for high risk of developing PC are strong but a proactive strategy, including a systematic second-look surgery plus HIPEC failed to improve survival, in comparison to an adequate surveillance. In addition, a recent French study have suggested that the addition of Oxaliplatin-HIPEC on the top of cytoreductive surgery does not influence both overall survival and disease-free survival but suggest that HIPEC with Oxaliplatin may be beneficial for patients with a medium PCI.^11^–^15^,^27^ Therefore, at the present time, the role of HIPEC in the treatment of PC is not clear, and long-term results have to be awaited to assess the role of prophylactic/adjuvant HIPEC.

As reported previously, the overall incidence of PC in our study was 34.3%. This could lead us to believe that one-third of patients might benefit from a proactive approach to avoid the development of PC. Nonetheless, in the group with synchronous PC (12%), 4% had distant metastasis, and of the 22.3% of patients with metachronous PC, 15.9% developed distant metastasis in the follow-up. Therefore, we deduced that prophylactic HIPEC might prevent the development of PC in 14.4% of patients. The other patients would also have distant metastasis.

Various randomized clinical trials currently in the recruitment phase are seeking to assess the oncological efficacy of prophylactic HIPEC in cT4 tumors considering this stage of disease as the only risk factor for the development of PC.^20^,^28^–^30^ In our study, although 22.3% of patients developed metachronous PC, only 6.1% of patients had metastasis at this site alone, and pT4 as a single risk factor was not shown to be an independent risk factor for the development of PC. Nonetheless, patients with pT4 tumors and peritoneal implants around the primary tumor and/or tumor perforation and/or who underwent emergency surgery had a higher rate of metachronous PC as a single site of metastasis than other patients.

## Conclusions

Considering pT4 tumor stage as the only risk factor for the development of PC in deciding whether to administer prophylactic or preemptive HIPEC would lead to unjustified overtreatment. Knowing that the role of HIPEC in the treatment of PC is not clear, we should wait for the results of the ongoing clinical trials before advocating either prophylactic or preemptive strategies for managing tumors in patients with various risk factors for developing PC.
